# Intersections of Substance Use, Overdose Risk, and Intimate Partner Violence: A Dyadic Approach to Violence Prevention in Criminal Legal Settings

**DOI:** 10.21203/rs.3.rs-6676375/v1

**Published:** 2025-05-26

**Authors:** Dawn Goddard-Eckrich, Phillip Marotta, Nishita Dsouza, Mingway Chang, Elwin Wu, Ariel Richer, Timothy Hunt, Alissa Davis, Anindita Dasgupta, Jennifer Hall, Sherna Alexander, Kacey-Ann Cockett, Nabila El-Bassel, Louisa Gilbert

**Affiliations:** Columbia University; Washington University in St. Louis; Columbia University; Columbia University; Columbia University; Columbia University; Columbia University; Columbia University; Columbia University Mailman School of Public Health, Department of Sociomedical Sciences; Columbia University; Columbia University; Columbia University; Columbia University; Columbia University

**Keywords:** Actor-Partner Interdependence Model, IPV, Couples, drug use, criminal legal system

## Abstract

**Background:**

When examining the relationship between intimate partner violence (IPV) and substance use, most studies have focused exclusively on individual-level correlates without considering cross-partner associations. Given the bidirectional nature of IPV within intimate relationships, a dyadic (couple) approach may assist in gaining a more precise understanding of the complex interrelationships between IPV, substance use, and overdose risks among couples.

**Methods:**

We employed the Actor-Partner Interdependence Model (APIM) using baseline data from a randomized controlled trial to examine how perpetration and experience of IPV in the past three months may be associated with substance use and non-fatal overdose risks among men in community supervision programs in New York City and their intimate partners (N=412 participants; 212 male and 196 female). The actor and partner effects were estimated using a multilevel logistic regression model with membership of a couple as a random effect for partner dependency, adjusting for key demographic factors.

**Findings:**

Over one-third of the participants (n=157, 38.1%) reported experiencing IPV and perpetrating IPV (n=149, 36.2%). Female participants who had been in the ER due to drugs or alcohol were more likely to report experiencing IPV (OR=2.62, 95% CI=1.02-6.88, p=0.046) and perpetrating IPV (OR=2.41, 95% CI=1.02-5.73, p=0.046) than those who had not been in the ER. Male participants who had been in the ER due to drugs or alcohol were also more likely to report perpetrating IPV (OR=3.07, 95% CI=1.31-7.16, p=0.010). Male participants who had experienced overdose were more likely to report perpetrating IPV than those who had not (OR=2.90, 95% CI=1.20, 7.01, p=0.018). No partner effects of overdose risks were significantly associated with participants’ reports of IPV.

**Implications for D&I Research:**

Our findings suggest a complex relationship between IPV and substance use behaviors, with primarily actor effects, but no significant partner effects. Understanding these relationships is important for developing integrated interventions that address both IPV and substance use risks among tice-involved populations. Such approaches may help address racial health inequities in drug overdose rates among non-Hispanic Black and Latinx populations, who are disproportionately impacted by the criminal legal system due to racialized drug laws and policing.

## Introduction

### Overview

Recent research shows strong associations between experiences of intimate partner violence (IPV) and experiences of non-fatal drug overdose among different populations of women (1-3), including women in community supervision programs (CSPs). High rates of physical and sexual IPV (1, 4, 5) may contribute to the risks of overdose. After release from jail or prison, the risk of death from overdose is 12.7 times higher among men in the US than in the general population (6), highlighting the need to understand the associations between substance use disorder and IPV, especially among men in the criminal legal system. To date, no studies have examined the association between IPV (either experiencing or perpetrating) and non-fatal overdose among men in the criminal legal system.

A systematic review by (7) highlighted the increased overdose risk associated with criminal legal involvement. Addressing overdose among criminal legal-involved individuals is crucial for reducing racial health disparities, as non-Hispanic Black and Latinx populations are disproportionately affected by overdose due to racialized drug laws and policing (8). Research on the relationships between IPV within couples and overdose risk among criminal legal-involved men and their partners is needed to address these co-occurring public health issues.

### Experiencing and perpetrating IPV behaviors

IPV affects approximately one in three women and one in four men during their lifetime (9). While research has often focused on male perpetration and female victimization in heterosexual relationships, studies in specific contexts have shown that female-initiated violence can also occur at significant rates (10-12). Most female-to-male violence occurs within relationships characterized by bidirectional violence (13, 11, 14). However, the impact of this violence is not symmetrical; women are more likely to experience severe injuries, fear, and controlling behaviors from male partners than vice versa (13, 15).

### IPV and Substance Use: Moving toward a dyadic approach

Alcohol use disorders are most strongly associated with IPV perpetration, because alcohol impairs cognitive functioning, emotion regulation, and impulse control (16-20). Cocaine and methamphetamine use also increases IPV perpetration risk through similar disinhibition mechanisms and by inducing paranoia and aggression (21, 22).

The relationship between substance use and IPV was bidirectional. Substance use can increase IPV perpetration risk; conversely, IPV victimization can lead to increased substance use as a form of self-medication for physical and psychological trauma (23-25). While considerable research has examined how men's substance use contributes to IPV perpetration, women's substance use can also be associated with both victimization and perpetration, although these pathways are less researched (26). Most studies on IPV and substance use have focused exclusively on individual-level correlates, neglecting cross-partner associations (27, 28). The actor-partner interdependence model (APIM) offers a more nuanced approach by examining both actor effects (how one's behavior predicts one’s own actions) and partner effects (how one's behavior influences one’s partner) (29). Another study found that veterans' drug abuse led to both their own IPV perpetration and their partners’ perpetration, although partner effects were not always reciprocal (30).

#### Study Aims and Hypotheses

To date, few studies have examined the effects of co-occurring IPV and non-fatal overdose at the couple level, with a scope for IPV that includes both victimization and perpetration. To our knowledge, this is the first study to apply the Actor-Partner Interdependence Model to examine both experiencing and perpetrating intimate partner violence among heterosexual couples where one partner is involved in the criminal legal system. While previous research has employed APIM to study other health behaviors among criminal legal-involved populations (Davis et al., 2020; Marotta et al., 2021a), none has specifically focused on the bidirectional nature of intimate partner violence and its association with substance use and overdose risk in this vulnerable population.

With mounting evidence that IPV may be a risk factor for overdose, there is an urgent need to employ dyadic approaches, such as APIM, to identify patterns of how perpetrating and/or experiencing IPV may contribute to the risk of drug overdose in this population. This investigation is particularly relevant for those involved in the criminal legal system, given the overlap between racial health disparities, IPV, and overdoses for the population. To address this gap, this study employs APIM to examine how perpetration and experience of IPV may be associated with non-fatal overdoses among men in community supervision programs in New York City and their intimate partners. This study hypothesizes that non-fatal overdoses and substance use disorders are significantly associated with an increased likelihood of intimate partner violence (IPV) perpetration for both male and female offenders. Specifically:

(H1) Male and female actors who experienced non-fatal overdoses and substance abuse had a significantly higher likelihood of self-reporting for a history of experiencing and/or perpetrating IPV (actor effects).

(H2) Male partners who experienced non-fatal overdose and substance abuse had a significantly higher likelihood of female actors’ self-reports for a history of experiencing and/or perpetrating IPV (partner effects).

(H3) Female partners who experienced non-fatal overdose and substance abuse had a significantly higher likelihood of male actors reporting a history of experiencing and/or perpetrating IPV (partner effects).

### Framework

The present study employs the Actor-Partner Interdependence Model (APIM) as an analytical framework. This model is based on a dyadic conceptualization of health outcomes. Decades of research have linked the individual health outcomes of relationship partners to both the actions of the other partner and the functioning of the overall relationship. For example, individuals tend to benefit from mutually satisfying committed relationships because they are correlated with improved health outcomes and decreased harmful behaviors for both individuals in the relationship (31). The health outcomes of individuals within a relationship are thought to be interdependent and better understood within the context of the dyad. A dyad model allows the researcher to better recognize a partner's effects on an individual's health, identify any asymmetrical influences in which one partner has more influence on the health outcomes of both within the relationship, and examine both experiencing and perpetrating behaviors. However, using APIM allows for the operationalization of the dyad theoretical framework through the determination of the nature and effect of the patterns of interdependence within relationships (32).

### Intervention Design & Study Procedures

Project Protect and Connect, also known as Project PACT, is a couple-focused HIV/STI prevention intervention that includes a randomized controlled trial investigating its efficacy in reducing sexual risk behavior. It was conducted between July 11, 2013, and May 17, 2016. In the current study, we conducted a secondary analysis using baseline data from this RCT. A detailed description of the study methods and sample characteristics is provided by (33). All study participants provided informed consent, and the study activities were approved by the Institutional Review Board at Columbia University.

Two-hour PACT intervention sessions were delivered in a community supervision program setting. Core elements of the intervention (e.g., couple-based HIV testing and referral, problem-solving skills, strategies for reducing risky sexual behavior) are described elsewhere (33, 34). The control arm consisted of individual rapid HIV/STI testing with pre- and post-test counseling and referrals to HIV/STI treatment and other health and/or social services. Randomized couples were randomized to either the intervention or control arm using a computer-generated randomization algorithm to balance the number of couples per study arm via an adaptive, biased-coin procedure (33). Assessments were performed at baseline, three, six, and 12-month follow-up post-intervention. The investigators were blinded to the treatment assignment until the final 12-month follow-up assessment.

### Participant Eligibility & Sample Recruitment

The Columbia University graduate research assistants recruited participants from Community Correction Provider (CCP) locations in New York City. Eligibility screening included couples who met all of the following criteria: 1) both partners age 18 or older, 2) both partners identified each other as their primary sexual partner, 3) both partners reported a relationship duration of at least three months, 4) at least one partner reported having had condomless vaginal and/or anal intercourse with the primary sexual partner in the past 90 days, 5) at least one partner reported exposure or suspected their partner of exposure to an outside HIV risk (e.g., engaged in condomless sex with a different partner, shared syringes, tested positive for an HIV/STI) in the past year, 6) both partners reported that they plan to stay in a relationship with each other for at least another year, 7) the male partner reported either use of illicit drugs or binge drinking (i.e., consumed five or more alcoholic beverages on a single occasion) in the past 90 days, or attended substance abuse treatment in the past 90 days, 8) the male partner was mandated to community supervision, alternative to incarceration (ATI) or probation verified by court records (33).

Participants were ineligible if they had cognitive or psychiatric impairments, were not proficient in English, or could not provide informed consent. In addition, 27 participants who reported having an order of protection against or felt unsafe completing the intervention with their partner, did not have a physical mail address, or lived more than 90 minutes from New York City were ineligible to participate. Participants were reimbursed up to $265 to complete all study procedures. The final analytical sample consisted of 206 heterosexual couples (412 individuals, 212 male and 196 female participants) with complete baseline data on the measures of interest (33).

### Measures

#### Sociodemographics:

Participants’ age, race/ethnicity (African American/Black or Hispanic/Latinx), education level (high school degree or higher/less than high school degree), marital status (single/married), employment status (employed/unemployed), homelessness in the past 90 days (yes/no), and food insecurity in the past 90 days (yes/no).

#### Binge Drinking -

Participants reported whether they had ever consumed five or more alcoholic drinks (for males) or four or more alcoholic drinks (for females) within a six-hour period (yes/no). This lifetime measure was used to capture patterns of problematic alcohol use that might be associated with IPV, which is consistent with the CAGE assessment timeframe.

#### Alcohol Misuse -

Participants responded to the four-item alcohol misuse CAGE questionnaire. Researchers categorized participant responses according to a cutoff of ≥ 2, which is considered clinically significant (35). Internal consistency in this sample was high (α = .95) (36, 37).

#### Illicit Drug Use -

Participants reported lifetime use of heroin, cocaine, crack, speedball (a heroin and cocaine combination), methamphetamines, stimulants, non-prescribed tranquilizers, non-prescribed opiates, ecstasy, hallucinogens, and other drugs that were not prescribed. Participants who reported previous use of these substances were categorized as having used illicit drugs. Lifetime measures were used to maintain consistency with the DAST-10 assessment timeframe and capture patterns of drug use that might be associated with IPV.

#### Overdose -

Participants reported any lifetime overdose experience (yes/no) along with lifetime emergency room treatment for a drug or alcohol use-related problem (i.e., overdose, loss of consciousness, and injuries caused by drugs or alcohol) (yes/no). While we acknowledge the limitations of lifetime measures, they were chosen to ensure sufficient statistical power for analysis, as recent overdose events were relatively rare in this sample.

#### Problematic drug use -

Participants responded to the DAST-10 questionnaire, a measure of problematic illicit or non-prescribed drug use (38). Researchers coded any problematic substance use with a cutoff value of ≥ 3. Good internal consistency was demonstrated in this sample (α = .88).

#### Intimate Partner Violence (IPV) -

Experience and perpetration of IPV were assessed using a 16-item abbreviated version of the Revised Conflict Tactics Scale (CTS2) (39). Participants reported any incidents of psychological, physical, or sexual violence by their intimate partner in the previous three months. They also reported perpetration of these acts of violence against intimate partners during the same timeframe. Psychological violence included items such as insulting or swearing a partner; physical violence included items such as pushing, shoving, or slapping; and sexual violence included items such as using force or threats to have sex. The researchers assessed internal consistency with Cronbach's alpha (0.71 for experiencing IPV and 0.65 for perpetrating IPV). The lower alpha for perpetration may be due to 1) participants' discomfort in admitting perpetration and 2) gender differences in reporting patterns.

### Analysis

Descriptive statistics for experiencing and perpetrating IPV were reported by sex. We also reported descriptive statistics for sociodemographic variables and substance use variables by gender and experience or perpetration of IPV. The chi-square test or t-test was used to test differences in characteristics between those who did and did not report IPV.

To examine the actor and partner effects of substance use associated with experiencing and perpetrating IPV, we employed a multilevel logistic regression model with couples as random effects, to account for dependency within dyads. We analyzed experiencing and perpetrating IPV as separate outcome variables in distinct models. Each model included participants' reports and their partners' reports on one substance use variable at a time (to avoid multicollinearity between potentially correlated substance use measures), gender (coded male = 1 and female = 0), and interaction terms between gender and participant reports.

The parameters associated with participants' reports represented the effects of substance use on female participants' IPV (female actor effect). We determined the actor effect of substance use on male participants' IPV by combining the parameters associated with participants' reports and the interaction term between sex and participants' reports. Similarly, we determined partner effects by analyzing how a partner's substance use affected the participant's IPV outcomes.

Our primary models were adjusted for age and study arm assignment. We conducted sensitivity analyses, including additional covariates (ethnicity, education, and food security), to assess the robustness of our findings. Odds ratios (OR) were reported as actor and partner effects, and statistical significance was assessed using the associated 95% confidence interval (CI) and p-value at the level of 0.05. All statistical analyses were performed using Stata 15.1

In total, we estimated 12 separate models: six models examining the associations between different substance use measures and experiencing IPV and six models examining associations with perpetrating IPV. This approach allowed us to examine each substance use variable separately, while avoiding multicollinearity issues that would arise from including multiple correlated substance use measures in a single model.

## Results

The data for this analysis were drawn from baseline assessments of 412 participants (196 female and 216 male) comprising 206 heterosexual couples. All male participants were involved in community supervision programs and reported binge drinking, illicit drug use, and/or attending drug treatment in the past 90 days according to the eligibility criteria. Table 1 presents the prevalence of experiencing and perpetrating IPV in the past three months among the study sample by gender. More than one-third of the participants (n = 157, 38.1%) reported experiencing IPV. A similar prevalence of IPV perpetration has also been reported (n = 149, 36.2%). There was no statistically significant difference in the prevalence of IPV between female and male participants. For example, 76 (38.8%) female and 81 (37.5%) male participants reported experiencing IPV, and 72 (36.7%) female and 77 (35.7%) male participants reported perpetrating IPV.

Regarding substance use patterns, the lifetime prevalence of illicit drug use was reported to be 56.1% in females and 63.4% in males. Binge drinking was reported in 46.4% of females and 55.6% of males. Problematic drug use (DAST ≥ 3) was indicated in 45.1% of females and 50.7% of males, whereas problematic alcohol use (CAGE ≥ 2) was present in 30.6% of females and 36.2% of males. A history of overdose was reported by 12.8% of females and 13.4% of males, and emergency room visits due to drug or alcohol problems were reported by 16.8% of females and 16.7% of males.

Tables 2a and 2b present the sociodemographic characteristics of the full sample by sex. The mean age was 35.1 years for female participants and 35.7 years for male participants. Most participants were African American/Black (69.9% of females and 77.3% of males), followed by Hispanic/Latinx (19.4% of females and 19.4% of males). Educational attainment was similar across genders, with 67.3% of females and 64.4% of males reporting high school education or higher. Most participants were unmarried (59.7% of females and 57.9% of males) and unemployed (70.4% of females and 76.4% of males). Food insecurity was reported by 41.3% of females and 45.4% of males, whereas homelessness in the past 90 days was reported by 8.7% of females and 12.0% of males. In the group of male participants, 71.9% (n = 100) who did not perpetrate IPV completed high school, compared to 50.7% (n = 39) of who reported perpetration of IPV. A difference was also found in food security between female participants who did not report perpetration of IPV (n = 44, 35.5%) and those who did report perpetration of IPV (n = 37, 51.4%).

Tables 3a and 3b show the bivariate associations between IPV and substance use when analyzing data at the individual level divided by male and female participants. In Table 3a (experiencing IPV), significant substance use differences emerged between those who did and did not report experiencing IPV. Among female participants, those experiencing IPV were significantly more likely to report emergency room visits due to drugs or alcohol (23.7% vs. 12.5%, p < 0.05) and problematic drug use as measured by DAST ≥ 3 (60.8% vs. 36.1%, p < 0.01). For male participants, experiencing IPV was associated with multiple substance use indicators including emergency room visits due to drugs or alcohol (23.5% vs. 12.6%, p < 0.05), problematic drug use (63.0% vs. 43.6%, p < 0.01), lifetime binge drinking (64.2% vs. 50.4%, p < 0.05), and problematic alcohol use as measured by CAGE ≥ 2 (45.5% vs. 30.9%, p < 0.05).

Table 3b reveals even stronger associations between substance use and perpetrating IPV, particularly for male participants. Among females, perpetrating IPV was significantly associated with problematic drug use (58.0% vs. 38.7%, p < 0.01) and problematic alcohol use (39.7% vs. 25.4%, p < 0.05). For male participants, all six substance use measures showed significant associations with IPV perpetration: lifetime overdose experiences (20.8% vs. 9.4%, p < 0.05), emergency room visits due to substances (26.0% vs. 11.5%, p < 0.01), illicit drug use (74.0% vs. 57.6%, p < 0.05), problematic drug use (65.8% vs. 42.8%, p < 0.01), binge drinking (67.5% vs. 48.9%, p < 0.01), and problematic alcohol use (46.7% vs. 30.4%, p < 0.05). These bivariate findings demonstrate pervasive associations between substance use and IPV, with males showing particularly strong and consistent relationships between substance use behaviors and IPV perpetration.

Table 4 shows that female participants who had been in the ER due to drugs or alcohol were more likely to report experiencing IPV than those who had not been in the ER (OR = 2.62, 95% CI = 1.02–6.88, p = 0.046). Both female and male participants with DAST scores of 3 and above were more likely to report experiencing IPV in their lifetime than their male counterparts (OR = 3.89, 95% CI = 1.69-9.00, p = 0001 for females; OR = 3.07, 95% CI = 1.36, 6,96, p = 0.007 for males). No significant associations were found between their partners’ substance use behavior and the participants’ experience of IPV.

Table 5. details perpetration among female participants, as being in the ER due to drug or alcohol (OR = 2.41, 95% CI = 1.02–5.73, p = 0.046), DAST scores equal to or greater than 3 (OR = 2.10, 95% CI = 1.07–4.14, p = 0.032), and binge drinking (OR = 1.94, 95% CI = 1.01, 3.73, p = 0.048) were significantly more likely to report IPV perpetration. Of the six multilevel models for male participants, five substance use variables were significantly associated with greater odds of reporting perpetrating IPV: overdose (OR = 2.90, 95% CI = 1.20, 7.01, p = 0.018), being in the ER due to drug or alcohol (OR = 3.07, 95% CI = 1.31–7.16, p = 0.010), illicit drug use (OR = 2.25, 95% CI = 1.09–4.63, p = 0.028), DAST score equal to or greater than 3 (OR = 2.36, 95% CI = 1.18–4.70, p = 0.015), and binge drinking (OR = 2.41, 95% CI = 1.25–4.63, p = 0.008). None of the partners’ substance use behaviors were significantly associated with participants’ reports of perpetration of IPV.

## Discussion

Our results revealed significant actor effects, where one's own substance use was associated with both experiencing and perpetrating IPV. For men under community supervision, problematic drug use was associated with experiencing IPV, while multiple substance use measures (overdose, emergency room visits, illicit drug use, problematic drug use, and binge drinking) were associated with perpetrating IPV. For women partnered with criminal legal-involved men, emergency room visits due to substance use and problematic drug use were associated with both experiencing and perpetrating IPV.

Our findings identified significant sex differences in IPV and substance use associations. For women, experiencing IPV was significantly associated with emergency room visits and problematic drug use as measured by the DAST, whereas for men, experiencing IPV was only linked to problematic drug use. This suggests that women who experience IPV may use substances as coping mechanisms following victimization (23, 25). Notably, we found no significant partner effects in our models examining IPV victimization, indicating that one's own substance use behaviors, rather than that of their partner, predicted their IPV experiences.

For IPV perpetration, we found strong actor effects, but no significant partner effects. Men's perpetration was significantly associated with five substance use measures: overdose, emergency room visits, illicit drug use, problematic drug use, and binge drinking. Perpetration by women was associated with emergency room visits, problematic drug use, and binge drinking. These findings highlight substance use as a risk factor for IPV perpetration, potentially reflecting substance-induced disinhibition or underlying trauma contributing to both behaviors (16).

Women experiencing IPV often face barriers in accessing harm reduction and treatment resources, including controlling behaviors from partners and limited healthcare access (4, 3). The criminal legal system creates additional health inequities and barriers for overdose prevention. In the context of IPV, intimate partners may impede engagement with supervision requirements and inadvertently fuel mass supervision systems. Previous research using this dataset has demonstrated that couple-focused interventions can improve outcomes despite IPV exposure (3, 40). Our Actor-Partner Interdependence Model also revealed strong actor effects but no significant partner effects, suggesting one's own substance use behaviors—rather than their partner's—predict involvement with family violence. This pattern extends previous APIM analyses from previous analysis on the PACT project, which similarly found significant actor effects for injection drug use (41) and sexual HIV risk behaviors (42).

Future research should explore evidence-based violence reduction strategies, including mental health treatment, conflict resolution skills, and appropriate involvement in law enforcement during conflicts, as well as develop couple-focused interventions like adapting Project PACT to address overdose prevention within intimate partnerships, focusing on relationship communication, couple satisfaction, and trust as key mechanisms for behavioral change. Research is also needed to interrupt contexts where IPV occurs, as violence often occurs when partners are under the influence of substances. People who perpetrate violence may use substances during heightened emotional states without ensuring naloxone access, and may exclude partners from overdose prevention planning. Understanding how violence perpetration affects drug use behaviors is critical. Perpetrators may be less likely to engage in harm reduction practices during emotional distress and may disrupt coordinated efforts to reduce drug use. Further research should explore evidence-based violence reduction strategies, including mental health treatment, conflict resolution skills, and appropriate involvement in law enforcement during conflicts.

**Figure 1:**
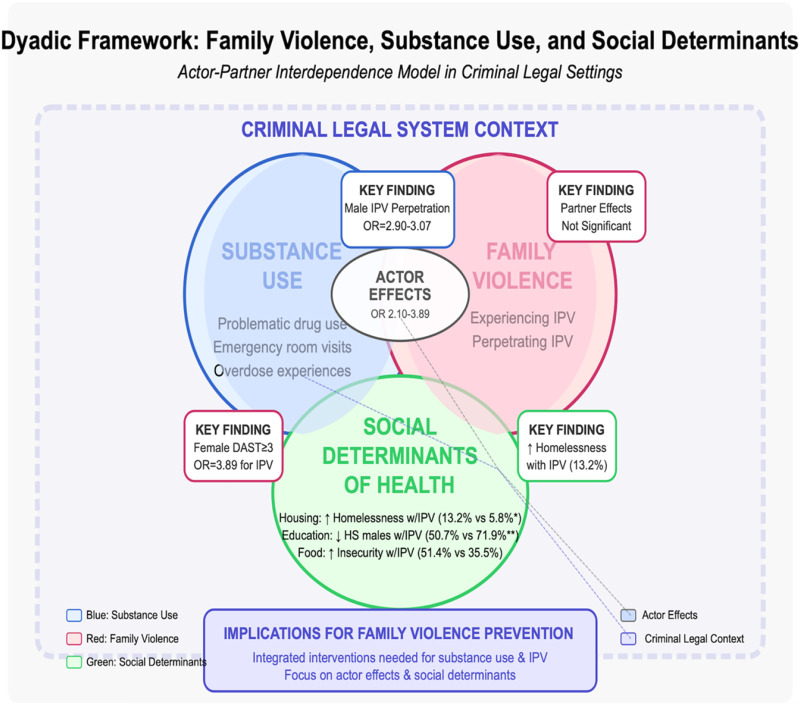
Legend not included with this version.

Our findings and dyadic framework (Fig. 1) also illustrates how social determinants of health (sdoh) shape the dynamics of family violence and substance use among criminal legal-involved couples. The observed associations between housing instability, food insecurity, and IPV align with (43) research on how basic needs insecurity shapes health outcomes and (44) work demonstrating how stressful life events exacerbate both substance use and intimate partner conflict. The educational disparities we identified among male perpetrators align with findings from other criminal legal-involved populations where socioeconomic factors predicted both substance use and risk behaviors (40). These social determinants create a web of vulnerability where substance use and family violence become mutually reinforcing, supporting previous work on the intersectional nature of these challenges (1).

These findings underscore the need for integrated approaches that simultaneously address substance use and IPV among criminal legal involved couples. Interventions should target both individual substance use behavior and relationship dynamics to effectively reduce IPV and substance-related harm in this vulnerable population.

## Limitations

This study has several limitations that warrant consideration. First, our cross-sectional analysis, using only baseline RCT data, prevented causal inferences. The temporal relationship between substance use and IPV cannot be established with certainty, as substance use measures captured lifetime experiences, while IPV measures focused on the past three months. Second, self-reported data introduce potential recall and social desirability bias that could result in underreporting of both substance use and IPV. This may be particularly relevant for IPV perpetration, which shows a lower internal consistency reliability. Third, our sample selection criteria may have introduced biases that limit generalizability. Male participants were selected based on community supervision involvement and substance use, and couples were included based on HIV risk criteria, potentially resulting in a sample with higher rates of both substance use and IPV than those of the general population. Fourth, we examined each substance use variable in separate models to avoid multicollinearity; however, this approach does not account for the common co-occurrence of different substance use patterns. Finally, conclusions should be limited to heterosexual couples in which the male partner is involved in the criminal legal system in New York City. The data were collected between 2013–2016, and patterns of substance use and overdose risk have evolved substantially with the changing opioid crisis, particularly with the increased fentanyl prevalence in recent years.

## Conclusion

This study addresses an important gap in the literature by examining the dyadic interplay between IPV and substance use among heterosexual couples, where the male partner is involved in community supervision. These findings highlight the need for approaches that simultaneously address substance use, family violence, and the underlying social determinants that contribute to both, particularly within criminal legal settings that serve populations disproportionately affected by these intersecting challenges. As community supervision increasingly serves as an alternative to incarceration for people who use drugs, understanding the complex relationships between substance use, IPV, and criminal legal system involvement is becoming increasingly important for developing effective interventions and policies, that can help reduce the public health burden of both IPV and substance use-related harm among families for this population.

## Figures and Tables

**Table 1:** Prevalence of IPV by Gender

	Female Participant (n = 196)	Male Participant (n = 216)	Total (N = 412)
Experiencing any IPV	76 (38.8%)	81 (37.5%)	157 (38.1%)
Experiencing severe IPV	43 (21.9%)	34 (15.7%)	77 (18.7%)
Perpetrating IPV	72 (36.7%)	77 (35.7%)	149 (36.2%)
Perpetrating severe IPV	36 (18.4%)	36 (16.7%)	72 (17.5%)

**Table 2 a:** Sociodemographic characteristics by Experiencing IPV and by gender

	Experiencing IPV			
	Female Participant		Male Participant	
	No (n = 120)	Yes (n = 76)	No (n = 135)	Yes (n = 81)
Age	*36.2 (13.3)*	*33.0 (12.8)*	36.6 (12.7)	34.2 (11.8)
Ethnicity				
African American/Black	84 (70.0%)	51 (67.1%)	110 (81.5%)	56 (69.1%)
Hispanic/Latino	25 (20.8%)	13 (17.1%)	22 (16.3%)	20 (24.7%)
High school	78 (65.0%)	54 (71.1%)	92 (68.2%)	47 (58.0%)
Marital status				
Single	75 (62.5%)	42 (55.3%)	76 (56.3%)	49 (60.5%)
Married	35 (29.2%)	29 (38.2%)	49 (36.3%)	29 (35.8%)
Currently unemployed	86 (71.7%)	51 (67.1%)	105 (77.8%)	60 (74.1%)
Homeless in the past 90 days	*7 (5.8%)*	*10 (13.2%)*	15 (11.1%)	11 (13.6%)
No enough money for food in the past 90 days	46 (38.3%)	35 (46.1%)	58 (43.0%)	40 (49.4%)

* p < 0.05

** p < 0.01 by two-tailed t-test or Chi-squared test

**Table 2 b:** Sociodemographic characteristics by Perpetrating IPV and by gender

	Perpetrating IPV			
	Female Participant		Male Participant	
	No (n = 124)	Yes (n = 72)	No (n = 139)	Yes (n = 77)
Age	35.9 (12.9)	33.3 (13.6)	35.1 (12.3)	36.9 (12.6)
Ethnicity				
African American/Black	86 (69.4%)	49 (68.1%)	108 (77.7%)	58 (75.3%)
Hispanic/Latino	24 (19.4%)	14 (19.4%)	27 (19.4%)	15 (19.5%)
High school	86 (69.4%)	46 (63.9%)	100 (71.9%)**	39 (50.7%)**
Marital status				
Single	75 (60.5%)	42 (58.3%)	84 (60.4%)	41 (53.3%)
Married	40 (32.3%)	24 (33.3%)	49 (35.3%)	29 (37.7%)
Currently unemployed	82 (66.1%)	55 (76.4%)	110 (79.1%)	55 (71.4%)
Homeless in the past 90 days	10 (8.1%)	7 (9.7%)	15 (10.8%)	11 (14.3%)

**Table 3 a:** Bivariate association between IPV and substance abuse, breaking by gender

	Experiencing IPV: Female Participant		Experiencing IPV: Male Participant	
	No (n = 120)	Yes (n = 76)	No (n = 135)	Yes (n = 81)
Overdose: ever	13 (10.8%)	12 (15.8%)	16 (11.9%)	13 (16.1%)
In ER due to drug or alcohol: ever	**15 (12.5%)** *	**18 (23.7%)** *	**17 (12.6%)** *	**19 (23.5%)** *
Illicit drugs (excluding marijuana): ever	68 (56.7%)	42 (55.3%)	79 (58.2%)	58 (71.6%)
DAST > = 3 (Problematic drug use)	**43 (36.1%)** **	**45 (60.8%)** **	**58 (43.6%)** **	**51 (63.0%)** **
Binge drinking: ever	52 (43.3%)	39 (51.3%)	**68 (50.4%)** *	**52 (64.2%)** *
CAGE > = 2 (Problematic alcohol use)	28 (26.9%)	27 (36.5%)	**38 (30.9%)** *	**35 (45.5%)** *

* p < 0.05

** p < 0.01 by Chi-squared test

**Table 3 b:** Bivariate association between IPV and substance abuse, breaking by gender

	Perpetrating IPV: Female Participant		Perpetrating IPV: Male Participant	
	No (n = 124)	Yes (n = 72)	No (n = 139)	Yes (n = 77)
Overdose: ever	15 (12.1%)	10 (13.9%)	**13 (9.4%)** *	**16 (20.8%)** *
In ER due to drug or alcohol: ever	16 (12.9%)	17 (23.6%)	**16 (11.5%)** **	**20 (26.0%)** **
Illicit drugs (excluding marijuana): ever	67 (54.0%)	43 (59.7%)	**80 (57.6%)** *	**57 (74.0%)** *
DAST > = 3 (Problematic drug use)	**48 (38.7%)** **	**40 (58.0%)** **	**59 (42.8%)** **	**50 (65.8%)** **
Binge drinking: ever	51 (41.1%)	40 (55.6%)	**69 (48.9%)** **	**52 (67.5%)** **
CAGE > = 2 (Problematic alcohol use)	**28 (25.4%)** *	**27 (39.7%)** *	**38 (30.4%)** *	**35 (46.7%)** *

* p < 0.05

** p < 0.01 by Chi-squared test

**Table 4:** Actor-Partner Independence Models for Experiencing IPV on Substance Abuse as Independent Variable (IV): Odds ratio, 95% confidence intervals and p-values

	Outcome: Experiencing IPV					
	Model 1 IV: Overdose: ever(n = 411)	Model 2 IV: In ER due to drug or alcohol: ever (n = 411)	Model 3 IV: Illicit drugs (excluding marijuana): ever (n = 411)	Model 4 IV: DAST > = 3 (among drug users; n = 367)	Model 5 IV: Binge drinking: ever (n = 411)	Model 6 IV: CAGE > = 2 (among alcohol users; n = 341)
Female actor effects						
Female actor’s substance abuse	1.53 [0.54, 4.35] (p = 0.416)	2.62* [1.02, 6.88] (p = 0.046)	0.86 [0.40, 1.84] (p = 0.699)	3.89** [1.69, 9.00] (p = 0.001)	1.55 [0.76, 3.19] (p = 0.229)	1.73 [0.79, 3.89] (p = 0.186)
Male partner’s effects on female actors	1.59 [0.58, 4.35] (p = 0.366)	0.88 [0.34, 2.24] (p = 0.787)	1.24 [0.57, 2.72] (p = 0.592)	0.56 [0.25, 1.26] (p = 0.160)	0.63 [0.31, 1.29] (p = 0.208)	0.52 [0.24, 1.15] (p = 0.108)
Male actor’s effects						
Male actor and his female partner	1.44 [0.54, 3.79] (p = 0.465)	2.42 [0.97, 6.06] (p = 0.059)	2.08 [0.94, 4.58] (p = 0.070)	3.07** [1.36, 6.96] (p = 0.007)	2.01 [0.99, 4.08] (p = 0.054)	1.70 [0.80, 3.60] (p = 0.164)
Female partner’s effects on male partners	1.33 [0.47, 3.74] (p = 0.592)	1.26 [0.48, 3.30] (p = 0.644)	0.88 [0.42, 1.83] (p = 0.726)	0.73 [0.33, 1.62] (p = 0.441)	1.51 [0.75, 3.03] (p = 0.248)	1.66 [0.75, 3.67] (p = 0.211)

* p < 0.05

** p < 0.01

**Table 5:** Actor-Partner Independence Models for Perpetrating IPV on Substance Abuse as Independent Variable (IV): Odds ratio, 95% confidence intervals and p-values

	Outcome: Perpetrating IPV					
	Model 1 IV: Overdose: ever(n = 411)	Model 2 IV: In ED due to drug or alcohol: ever(n = 411)	Model 3 IV: Illicit drugs (excluding marijuana): ever (n = 411)	Model 4 IV: DAST > = 3 (among drug users; n = 367)	Model 5 IV: Binge drinking: ever(n = 411)	Model 6 IV: CAGE > = 2 (among alcohol users; n = 341)
Effect Type						
Female actor’s effect	1.04 [0.41, 2.66] (p = 0.924)	2.41* [1.02, 5.73] (p = 0.046)	1.21 [0.61, 2.40] (p = 0.578)	2.10* [1.07, 4.14] (p = 0.032)	1.94* [1.01, 3.73] (p = 0.048)	1.89 [0.91, 3.92] (p = 0.087)
Male partner’s effects on female actors	1.77 [0.73, 4.30] (p = 0.210)	0.71 [0.30, 1.69] (p = 0.437)	1.14 [0.56, 2.30] (p = 0.725)	0.71 [0.36, 1.37] (p = 0.315)	0.78 [0.41, 1.49] (p = 0.453)	0.99 [0.49, 2.01] (p = 0.980)
Effect Type						
Male actor effects	2.90* [1.20, 7.01] (p = 0.018)	3.07** [1.31, 7.16] (p = 0.010)	2.25* [1.09, 4.63] (p = 0.028)	2.36* [1.18, 4.70] (p = 0.015)	2.41** [1.25, 4.63] (p = 0.008)	1.81 [0.91, 3.58] (p = 0.089)
Female partner’s effects on male actors	0.63 [0.24, 1.71] (p = 0.367)	0.89 [0.36, 2.19] (p = 0.797)	0.92 [0.47, 1.78] (p = 0.796)	1.42 [0.73, 2.78] (p = 0.304)	0.86 [0.46, 1.63] (p = 0.651)	1.98 [0.96, 4.09] (p = 0.064)

* p < 0.05

** p < 0.01

## Data Availability

The data presented in this study are available on request from the corresponding author. The data are not publicly available due to privacy and ethical reasons.
